# Torsion of an Accessory Spleen Presenting as Acute Right Iliac Fossa Pain: A Case Report

**DOI:** 10.7759/cureus.88006

**Published:** 2025-07-15

**Authors:** Jamal Ouachaou, Aicha Driouich, Mohammed Sidayne, Fatimazahrae El Khettab, Zarrouki Youssef

**Affiliations:** 1 Anesthesiology and Intensive Care, Mohammed VI University Hospital of Marrakech, Faculty of Medicine and Pharmacy, Cadi Ayyad University, Marrakesh, MAR

**Keywords:** abdominal computed tomography (ct) scan, accessory spleen, acute abdomen, laparoscopic surgery, torsion

## Abstract

Accessory spleens are typically identified incidentally. Torsion of an accessory spleen, however, is an exceptionally rare cause of acute abdominal pain and presents a significant diagnostic challenge due to its non-specific clinical presentation. We report the unusual case of a young woman admitted to the emergency department for an acute abdomen, leading to the laparoscopic diagnosis of accessory spleen torsion. This case highlights the diagnostic difficulties encountered preoperatively and underscores the pivotal role of laparoscopy in both the diagnosis and treatment of this rare condition. A review of the literature on medical management is also included.

## Introduction

An accessory spleen is a congenital anatomic abnormality characterized by ectopic splenic tissue that does not fuse with the main splenic mass [1]. It is a common condition that has been noted in 10% of abdominal CT scans [2]. However, torsion of an accessory spleen is an extremely rare cause of acute abdominal pain. Only a few cases have been reported in the medical literature [3]. Its clinical presentation is non-specific, and in most cases, the diagnosis is made during surgery, even with the use of computed tomography [4]. Herein, we describe the rare case of a young patient admitted to the emergency department for an acute abdomen caused by torsion of an accessory spleen.

## Case presentation

A healthy 28-year-old woman, with no medical history, presented with acute right iliac fossa pain evolving over the past three days, described as sharp and persistent, and associated with nausea and vomiting. There is no urinary complaint and no menstrual delay; her last period was 10 days ago. On examination, she was mildly distressed and preferred to lie still. Abdominal palpation revealed tenderness in the right iliac fossa with guarding and rebound tenderness; Rovsing’s sign was positive. Vital signs showed a temperature of 38.1°C, heart rate of 94 bpm, blood pressure of 120/76 mmHg, respiratory rate of 18 breaths per minute, and oxygen saturation of 98% on room air. A pregnancy test, complete blood count (CBC), C-reactive protein (CRP), urinalysis, and abdominal-pelvic ultrasound were requested. Acute appendicitis was the leading diagnosis, with ovarian torsion and ruptured cyst considered in the differential. Laboratory analysis showed a white blood cell count of 6900 cells/µL, a low level of serum CRP (11 mg/L), and a normal HCG level (2 IU/L) (Table 1).

**Table 1:** Laboratory findings.

Test	Result (SI units)	Normal range (SI units)
Hemoglobin g/dl	10.50	8.4-11.2
Hematocrit %	0.43	0.40-0.54
White blood cells /µL	6900	4000-10000
Platelets cells/µL	350000	150,000-400,000
Urea (mg/L)	0,42	0.25-0.48
Creatinine µmol/L	80,3	60-110
Sodium mmol/L	142	135-145
Potassium mmol/L	4.2	3.5-5.0
Prothrombin ratio %	90	70-100
C-reactive protein (CRP) (mg/L)	11	<2
Human chorionic gonadotropin (HCG) (mIU/mL)	2	<5

**Figure 1:**
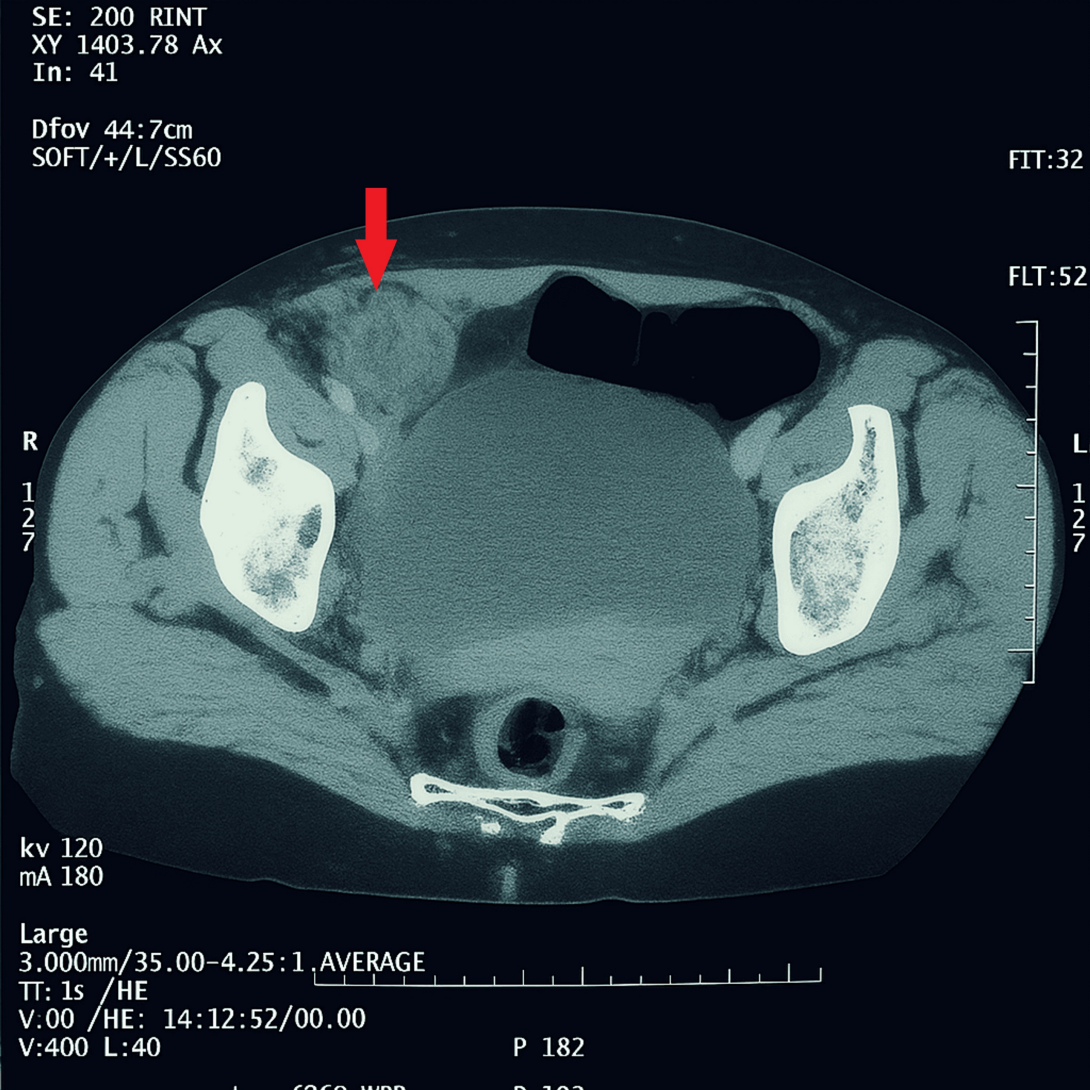
CT scan of the pelvic region showing a right laterocystic mass with a wall slightly enhanced by the contrast product (red arrow).

Ultrasonography (US) revealed a minimal hypoechoic effusion in the right iliac fossa. The spleen was normally located. An abdominal CT scan showed a heterogeneous mass in the right iliac fossa, which slightly enhanced (Figure 1).

**Figure 2:**
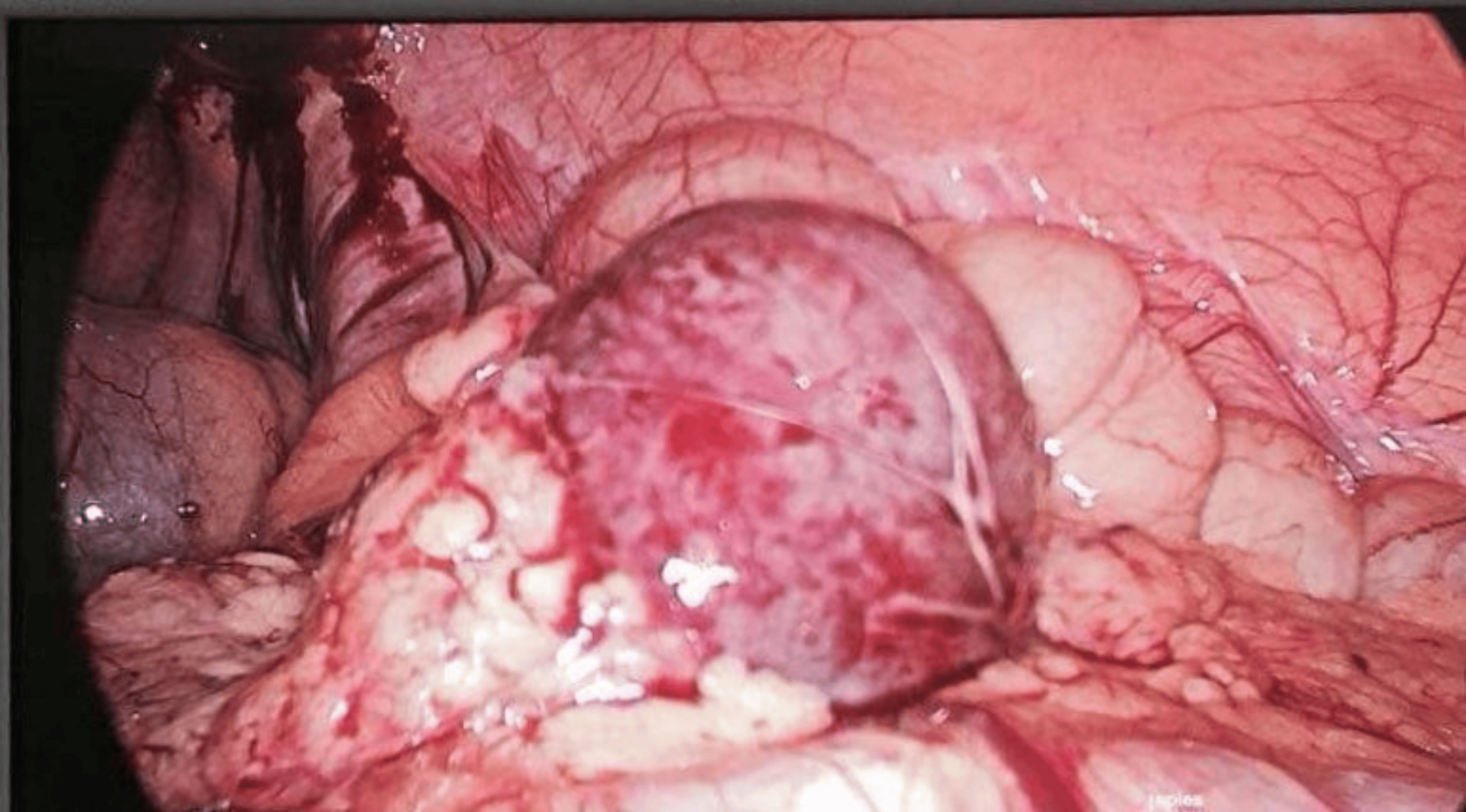
Laparoscopic view of the mass.

**Figure 3:**
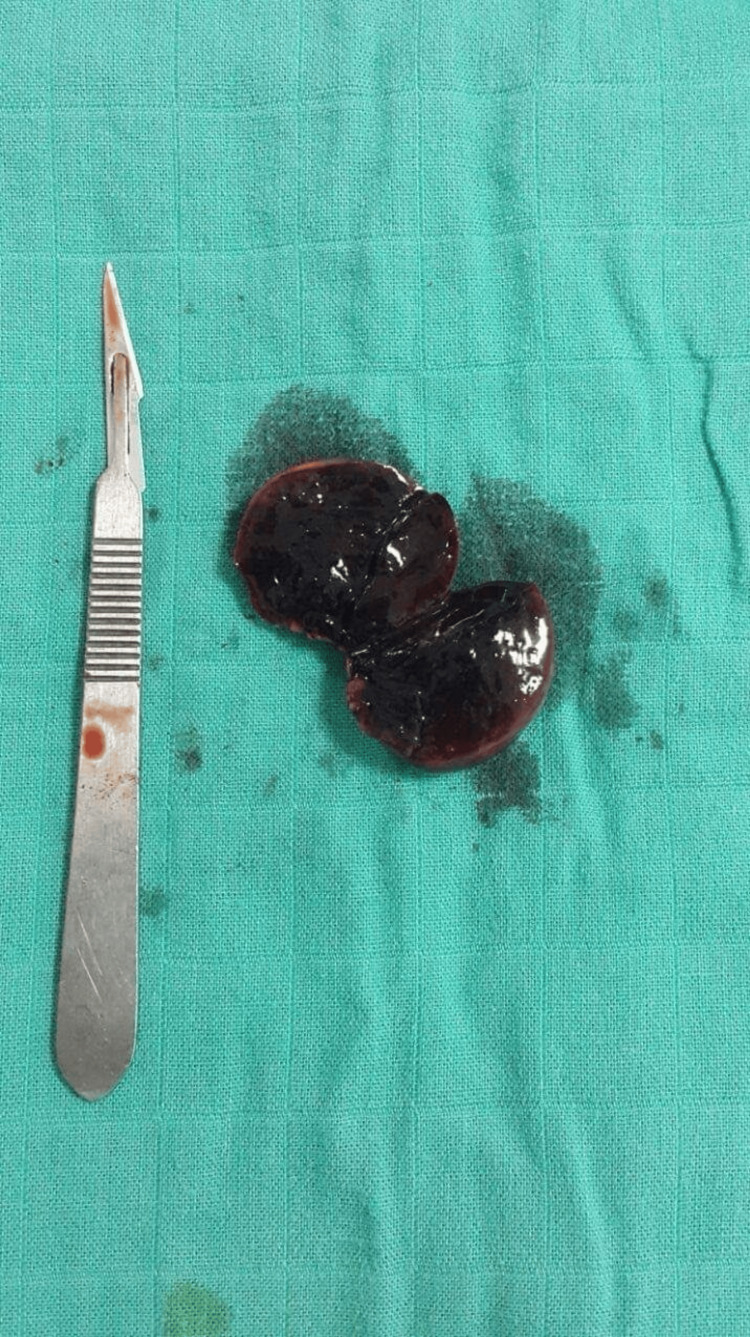
Necrotic accessory spleen

Based on the radiological findings, laparoscopic surgery was performed, revealing a 3 cm twisted mass on its pedicle (Figure 2), which was resected after clipping the pedicle (Figure 3).

Anatomopathological examination confirmed the diagnosis of an accessory spleen torsion. The postoperative course was uneventful. The patient got discharged four days after.

## Discussion

Accessory splenic torsion is a rare and often under-recognized cause of acute abdominal pain. Due to its non-specific clinical, biological, and radiological presentations, as well as its rarity, the diagnosis is most often made intraoperatively [5,6] .Patients generally arrive with acute or subacute abdominal pain, which is usually focused in the left upper quadrant, left flank, or even the lower abdomen, depending on where the accessory spleen is situated, nausea, vomiting, and low-grade fever.

In most cases, accessory spleens are clinically silent and can be discovered incidentally on abdominal imaging requested in the context of other pathologies. Complications are very rare and may include torsion, infarction, bleeding, and infection [7,8,9,10].

The spleen develops from mesenchymal tissue in the dorsal mesogastrium during the fifth week of gestation. Accessory spleens likely originate from mesenchymal remnants that fail to fuse with the main splenic mass. The most common site for accessory spleens is the splenic hilum, but other locations include the splenic vessels, omentum, and retroperitoneum [7,11,12].

US findings can vary, with the accessory spleen presenting as a hypoechoic or homogeneous parenchymal mass located in any quadrant of the abdomen. On enhanced CT scan, twisted accessory spleens typically appear as well-marginated, rounded masses that enhance heterogeneously depending on blood flow in the twisted pedicle. MRI has advantages over US and CT, not only for detecting the mass but also for evaluating its nature. However, MRI is not always feasible in emergency situations [7,10]. In this case, the CT scan was crucial in identifying an abdominal mass but could not definitively diagnose the etiology, reinforcing the challenge of preoperative diagnosis. The location in the right iliac fossa is also unusual, further complicating the clinical picture.

Nonetheless, preoperative diagnosis is often hypothetical. Laparoscopy with histological examination remains the gold standard for both diagnosis and treatment [10,13,14]. Laparotomy is usually reserved for complicated cases (e.g., peritonitis or abundant hemoperitoneum) or when a large accessory spleen cannot be extracted via laparoscopic surgery.

In this case, laparoscopy was the first-line intervention, providing both diagnosis and treatment. This situation differs from the torsion of a wandering spleen, where the diagnosis is more obvious due to the absence of the splenic compartment on radiological examination [11].

The surgical removal of an accessory spleen does not result in the immune deficiency seen in splenectomized patients with a single spleen, so postoperative vaccination is not indicated.

## Conclusions

While accessory spleens are common, torsion of an accessory spleen is a rare cause of acute abdominal pain in the emergency room. It is helpful for radiologists to be aware of the possibility of accessory spleen torsion so that the diagnosis can be suggested early, especially when an avascular mass is seen on the CT scan, and the normal spleen remains in place.

The definitive diagnosis is obtained in the operating room after anatomopathological examination.
